# Comparative Safety of Advanced Therapies for Crohn Disease

**DOI:** 10.1001/jamanetworkopen.2025.57922

**Published:** 2026-02-06

**Authors:** Soo-kyung Park, Dhruv Ahuja, Kuan-Hung Yeh, Sagar B. Patel, Shane W. Goodwin, Christopher Ma, Namrata Singh, Ashwin N. Ananthakrishnan, Vipul Jairath, Ronghui Xu, Siddharth Singh

**Affiliations:** 1 Division of Gastroenterology, Department of Medicine, University of California San Diego, La Jolla; 2 Department of Gastroenterology, Samsung Kangbuk Hospital, Sungkyunkwan University School of Medicine, Seoul, Korea; 3 Department of Medicine, Indira Gandhi Hospital, New Delhi, India; 4 Division of Biomedical Informatics, Department of Medicine, University of California San Diego, La Jolla; 5 London Health Sciences Centre Research Institute, London, Ontario, Canada; 6 Division of Gastroenterology and Hepatology, Cumming School of Medicine, University of Calgary, Calgary, Alberta, Canada; 7 Department of Community Health Sciences, University of Calgary, Calgary, Alberta, Canada; 8 Division of Rheumatology, University of Washington, Seattle; 9 Division of Gastroenterology, Massachusetts General Hospital, Boston; 10 Harvard Medical School, Boston, Massachusetts; 11 Division of Gastroenterology, Western University, London, Ontario, Canada; 12 Department of Epidemiology and Biostatistics, Western University, London, Ontario, Canada; 13 Division of Biostatistics and Bioinformatics, Herbert Wertheim School of Public Health and Human Longevity Science, University of California San Diego, La Jolla; 14 Division of Gastroenterology and Hepatology, Department of Medicine, Mayo Clinic in Arizona, Scottsdale

## Abstract

**Importance:**

With the availability of multiple classes of advanced therapies for the treatment of Crohn disease (CD), understanding the comparative safety of different therapies can inform treatment positioning.

**Objective:**

To compare the risk of serious infections, venous thromboembolism (VTE), and major adverse cardiovascular events (MACE) with different advanced therapies in patients with CD.

**Design, Setting, and Participants:**

This retrospective comparative effectiveness research study was conducted between January 1, 2016, and December 31, 2022, with a mean (SD) follow-up of 26.9 (2.4) months until July 1, 2024. Using an administrative claims database (OptumLabs Data Warehouse), commercially insured patients with CD, who initiated treatment with tumor necrosis factor-α (TNF) antagonists, anti-integrin agents (vedolizumab), interleukin (IL)-12/23p40 antagonists (ustekinumab), IL-23p19 antagonists (primarily risankizumab), or Janus kinase inhibitors (upadacitinib) between 2016 and 2022 and had follow-up for at least 1 year before and after treatment initiation, were included.

**Exposure:**

TNF antagonists vs anti-integrin agents (vedolizumab) vs IL-12/23p40 antagonists (ustekinumab) vs IL-23p19 antagonists (risankizumab) vs Janus kinase inhibitors (upadacitinib).

**Main Outcomes and Measures:**

The risk of serious infections, VTE, and MACE was compared with various advanced therapies through multinomial propensity score-based inverse probability treatment weighting, with propensity scores estimated through generalized boosted models, accounting for disease characteristics, health care utilization, comorbidities, and prior and concomitant medications, and through competing risk of mortality. Cause-specific hazard ratios (HRs) and 95% CIs for multiple treatment comparisons were calculated.

**Results:**

This study included 12 245 patients with CD (mean [SD] age, 46.5 [17.5] years; 6642 females [54.2%]), who were treated with TNF antagonists (n = 5274), vedolizumab (n = 2716), ustekinumab (n = 3544), risankizumab (n = 559), or upadacitinib (n = 152). Serious infection incidence rates ranged from 5.46 (95% CI, 4.86-6.07) to 9.02 (95% CI, 6.38-11.89) per 100 person-years across therapies. After adjusting for confounding variables, there were no statistically significant differences in the risk of serious infections across different agents, including between risankizumab and ustekinumab (HR, 1.14 [95% CI, 0.78-1.67]), risankizumab and TNF antagonists (HR, 1.00 [95% CI, 0.68-1.47]), or ustekinumab and TNF antagonists (HR, 0.88 [95% CI, 0.74-1.04]). The incidence of VTE (incidence rate, 0.90 [95% CI, 0.71-1.10] to 2.33 [95% CI, 1.06-3.82] per 100 person-years) and MACE (0.68 [95% CI, 0.51-0.85] to 1.49 [95% CI, 0.43-2.76] per 100 person-years) was low, without any significant differences across agents.

**Conclusions and Relevance:**

In this comparative effectiveness research study of patients with CD, no significant differences in the risks of serious infections, VTE, or MACE across various advanced therapies were found. These findings support clinical decision-making on choice of advanced therapies for most individual patients with CD to be driven primarily by comparative treatment effectiveness rather than driven by concerns of serious adverse events.

## Key Points

**Question:**

What is the comparative safety of different advanced therapies in patients with Crohn disease (CD)?

**Findings:**

In this comparative effectiveness research study of 12 245 patients with CD, treated with tumor necrosis factor-α antagonists, vedolizumab, ustekinumab, risankizumab, or upadacitinib and followed up over a mean 27 months, no clinically meaningful differences in the risk of key safety events, including serious infections, venous thromboembolism, and major adverse cardiovascular events, were found. The incidence of venous thromboembolism and major adverse cardiovascular events was low across all therapies.

**Meaning:**

The findings of this study suggest that the safety of different advanced therapies in patients with CD was comparable and should not be the primary driver of treatment positioning in most instances.

## Teaser

This comparative effectiveness research study examines the risk of key safety events associated with different advanced therapies in patients with Crohn disease.

## Introduction

Since the introduction of biologic therapies for inflammatory bowel disease (IBD) in the late 1990s, their use has expanded substantially, particularly among patients who exhibit inadequate response to conventional immunomodulatory treatments.^1,2^ For Crohn disease (CD), the therapeutic arsenal has progressed from tumor necrosis factor-α (TNF) antagonists to include agents such as vedolizumab, an anti-integrin monoclonal antibody, and ustekinumab, which targets the shared p40 subunit of interleukin (IL)-12 and IL-23.^3,4^ More recently, monoclonal antibodies directed against IL-23p19, have been approved.^5,6,7,8^ In parallel with these biologic agents, small molecule therapies—most notably upadacitinib, a selective Janus kinase (JAK) 1 inhibitor—have emerged as treatment options.^9^

While head-to-head randomized clinical trials (RCTs) and subsequent network meta-analyses inform the comparative efficacy of these agents, there is significant paucity of data on the comparative safety of these agents, a key factor in clinical decision-making for choosing the optimal therapy.^10,11^ Serious adverse events such as infections requiring hospitalization, venous thromboembolism (VTE), and major adverse cardiovascular events (MACE) are rare events for which RCTs are not adequately powered. Moreover, RCTs typically have a short duration of follow-up and may have a high degree of selection bias, excluding patients with significant comorbidities. Consequently, comparative safety of different advanced therapies is better informed through comparative observational studies. In a previous systematic review and meta-analysis of 20 comparative observational studies, Solitano et al^12^ observed that there were no significant differences in the risk of serious infections between vedolizumab and TNF antagonists in patients with CD, and ustekinumab may have been associated with a lower risk of serious infections compared with TNF antagonists and vedolizumab, offering a possible net benefit. With the availability of several newer non–TNF-targeting biologics and oral small molecule drugs, ongoing, well-designed comparative effectiveness studies are warranted to optimally inform risks associated with these agents. Moreover, with the recognition of a potentially higher risk of MACE and VTE with JAK inhibitors compared with TNF antagonists in older patients with rheumatoid arthritis, there is considerable interest in examining the risk of these rare events in patients with CD.^13^ Thus, we conducted a retrospective comparative effectiveness research study using a deidentified administrative claims database to evaluate and compare the risk of serious infections, VTE, and MACE in patients with CD who were treated with TNF antagonists, anti-integrin agents (vedolizumab), IL-12/23p40 antagonists (ustekinumab), IL-23p19 antagonists (primarily risankizumab based on timing of drug approval), and JAK inhibitors (upadacitinib).

## Methods

### Data Source

In this comparative effectiveness research study, we conducted a retrospective analysis of deidentified medical and pharmacy administrative claims from a large database, OptumLabs Data Warehouse.^14^ As this study used deidentified administrative claims data, it was deemed exempt from institutional review board oversight in accordance with US federal regulations (Common Rule), and the requirement to obtain informed consent was therefore waived. This study followed the Strengthening the Reporting of Observational Studies in Epidemiology (STROBE) and the International Society for Pharmacoeconomics and Outcomes Research (ISPOR) reporting guidelines.

### Study Population

We identified patients with CD who were new users of advanced therapies (TNF antagonists [infliximab, adalimumab, and certolizumab pegol], anti-integrin agents [vedolizumab], IL-12/23p40 antagonists [ustekinumab], IL-23p19 antagonists [risankizumab], and JAK inhibitors [upadacitinib]) between January 1, 2016, and December 31, 2022, with a mean (SD) follow-up of 26.9 (2.4) months. We included adult patients (aged 18-65 years) with (1) at least 1 diagnosis code for CD (*International Classification of Diseases, Ninth Revision *[*ICD-9*] 555.x or *International Statistical Classification of Diseases and Related Health Problems, Tenth Revision *[*ICD-10*] K50) prior to the index date (the date of the first filled prescription or infusion for advanced therapies of interest), either from an inpatient or outpatient visit; (2) continuous health plan enrollment with pharmacy benefits, with no prescription for a respective advanced therapy in the 12 months prior to the index date (new-user design); and (3) a minimum 12-month enrollment in a health plan after the index date; patients who received index therapy for less than 12 months and discontinued due to intolerance or nonresponse but still remained in the health plan were included. This combination of 1 or more diagnosis codes for IBD together with a prescription for an IBD-related medication has a positive predictive value of 78% for prevalent cases of IBD in the OptumLabs Data Warehouse.^15^ In a sensitivity analysis, we excluded patients with comorbid immune-mediated inflammatory diseases (IMIDs), including rheumatoid arthritis, psoriasis, psoriatic arthritis, and ankylosing spondylitis, for which these medications may be prescribed.

### Exposure and Comparator

The primary exposures of interest were classes of advanced therapies: TNF antagonists, anti-integrin agents (vedolizumab), IL-12/23p40 antagonists (ustekinumab), IL-23p19 antagonists (risankizumab), and JAK inhibitors (upadacitinib). To facilitate comparison across multiple drug classes, we classified each patient as being exposed to a specific class of advanced therapies at each time point, with treatment episode being our study’s unit of analysis. Patients could contribute to different exposure groups sequentially if they met criteria for being a new user for the other advanced therapy; patients exposed to multiple advanced therapies simultaneously were excluded. Patients who switched to another medication within the same class contributed to exposure time for that class of advanced therapy through the combined time of both.

### Outcome

The study’s outcomes of interest included serious infections, VTE, and MACE. Serious infections were identified by *ICD-9* or *ICD-10* codes in any first 5 diagnoses suggestive of an infection requiring hospitalization (positive predictive value of ≥80%).^16^ Serious infections were classified as being of gastrointestinal and extraintestinal origin to analyze differential risk; gastrointestinal infections, including abdominal and perianal abscesses, may have been more likely reflective of disease-related complications and indirectly related to treatment effectiveness, whereas extraintestinal infections were more likely reflective of treatment-related risks associated with systemic immune suppression. VTE was identified by deep venous thrombosis and/or pulmonary embolism during inpatient, emergency department, and/or an office visit, identified based on the top 5 *ICD-10* diagnosis codes related to the visit; a sensitivity definition focused only on events during an inpatient or an emergency department visit.^17^ MACE was identified by a composite of myocardial infarction, unstable angina, a cardiovascular revascularization procedure (including percutaneous coronary intervention or coronary artery bypass graft surgery), stroke (ischemic or hemorrhagic), or a transient ischemic attack as the primary discharge diagnosis during an inpatient or emergency department visit.^18^

### Covariates

We included the following baseline covariates (at the time of advanced therapy exposure or in the preceding 12 months): age, sex, race and ethnicity, calendar year, and smoking (demographics)^19^; obesity, hyperlipidemia, smoking, diabetes, coronary artery disease, prior hip or knee surgery, and overall burden of comorbidities based on the Elixhauser Comorbidity Index (comorbidities); all-cause hospitalization or emergency department visits, receipt of lower endoscopy, abdominal imaging, IBD-related surgery, and serious infections (health care utilization); and prior and concomitant exposure to immunomodulators, corticosteroids, and opiates; prior exposure to IBD-related advanced therapies; and prior use of statins, hormonal contraceptives, antiplatelet drugs, and anticoagulants (medication use). In the OptumLabs Data Warehouse, race and ethnicity are derived from health plan enrollment files and, for a subset of individuals, from electronic health record–linked administrative data, based on self-reported information if available. Race and ethnicity categories included Black, Hispanic, White, and other, which included American Indian or Alaska Native, Asian, Pacific Islander, and those not captured in predefined categories. We did not have access to individual patient medical records, endoscopy reports, or biochemical parameters.

### Statistical Analysis

We examined the incidence rate per 100 person-years of adverse events of interest after starting advanced therapies. To estimate this, patients were followed until they experienced the outcome of interest, disenrolled from a health care plan, or discontinued index therapy (absence of a refill for a period of >4 months) or until the study end point for follow-up (July 1, 2024). Events were attributed to index therapy if they occurred during the time that the patient was exposed to medication, prior to switching or stopping therapy.

To mitigate the confounding bias due to the observational nature of the data, we applied multinomial propensity score–based inverse probability weighting (IPW) for comparison of risk of events across multiple therapeutic groups.^20^ Confounders for propensity score modeling were identified based on both clinical expertise and univariate assessment, specifically their associations with the treatment assignment and the outcome of interest using univariate analyses. To evaluate associations with treatment groups, we fitted a multinomial logistic regression model including each covariate. Covariates with *P* < .10 were retained as potential confounders. Similarly, associations with the outcome were assessed using cause-specific Cox proportional hazards regression models, and variables with *P* < .10 were also flagged as potential confounders. Variables associated with both treatment assignment and the outcome of interest were considered confounders and included in the propensity score model. In addition, prior biologics exposure, concomitant use of steroids, hospitalizations in the preceding year, and a few outcome-specific variables were added based on clinical expertise. The outcome-specific variables included prior serious infections (for serious infections) and prior VTE (for VTE).

Multinomial propensity scores were estimated from a generalized boosted model using the R package twang, version 4.3.2 (R Project for Statistical Computing), a specialized toolkit for weighting and analyzing nonequivalent groups. IPW derived from the propensity scores was stabilized using the marginal probability of treatment assignment. To limit the influence of extreme weights, values were truncated to the range 0.1 to 10.0, when necessary. To assess the covariate balance, confounders with an absolute standardized mean difference of less than 0.1 were considered as balanced. As patients could contribute 1 or more episodes of new use (with an updated set of covariates), we used the robust sandwich variance estimator and calculated robust SEs for all estimates.^21^

To account for mortality as a competing risk, we defined the time to the first event of interest, the censoring event (disenrollment, treatment discontinuation, or study end), or death (as the competing event), starting from the treatment index date. Cause-specific hazard ratios (HRs) and 95% CIs for the outcomes were estimated using a Cox proportional cause-specific hazards regression model.^22^ This weighted model incorporated stabilized weights and adjusted for covariates that remained unbalanced after weighting if necessary. All statistical analyses were performed using R, version 4.3.2. A 2-sided *P* < .05 was considered statistically significant.

## Results

### Patient Characteristics

Our study’s cohort included a total of 12 245 patients with CD (mean [SD] age, 46.5 [17.5] years; 6642 females [54.2%] and 5603 males [45.8%]). Among these patients, 5274 were treated with TNF antagonists and followed up for a mean (SD) 28.0 (8.6) months, 2716 treated with anti-integrins and followed up for 27.4 (9.4) months, 3544 treated with IL-12/23p40 antagonists and followed up for 26.2 (9.5) months, 559 treated with IL-23p19 antagonists and followed up for 12.4 (3.9) months, and 152 treated with JAK inhibitors and followed up for 27.3 (8.7) months.

Baseline characteristics of patients are shown in Table 1. Of the total patients, 457 (3.7%) were Black, 475 (3.9%) were Hispanic, 8857 (72.3%) were White, and 2456 (20.1%) were of other race or ethnicity. Among patients treated with a TNF antagonist (mean [SD] age, 44.1 [17.7] years; 2775 females [52.6%]; 3729 White [70.7%]), 15 (0.3%) had received vedolizumab and 155 (2.9%) had received ustekinumab in the preceding 1 year, and 1756 (33.3%) were concomitantly receiving corticosteroids at the time of starting the TNF antagonist. Among patients treated with an IL-12/23p40 antagonist (mean [SD] age, 46.7 [16.9] years; 1965 females [55.4%]; 2605 White [73.5%]), 1222 (34.5%) had received a TNF antagonist in the preceding 1 year, and 1024 (28.9%) were concomitantly receiving corticosteroids at the time of starting ustekinumab. Among patients treated with a JAK inhibitor (mean [SD] age, 48.3 [15.6] years; 102 females [67.1%]; 119 White [78.3%]), 48 (31.6%) had received a TNF antagonist in the preceding 1 year, and 64 (42.1%) were concomitantly receiving corticosteroids at the time of starting upadacitinib. Concomitant immunomodulator use ranged from 7.2% in IL-23p19 antagonists to 20.1% in TNF antagonists, consistent with patterns shown in Table 1.

**Table 1.** Baseline Characteristics of Patients With Crohn Disease Treated With Advanced Therapies

Variable	Patients, No. (%)					
	Overall (N = 12 245)	TNF-α antagonists (n = 5274)	Anti-integrin (n = 2716)	IL-12/23p40 antagonists (n = 3544)	IL-23p19 antagonists (n = 559)	JAK inhibitors (n = 152)
Age, mean (SD), y	46.5 (17.5)	44.1 (17.7)	50.2 (17.7)	46.7 (16.9)	49.2 (16.0)	48.3 (15.6)
Sex						
Female	6642 (54.2)	2775 (52.6)	1482 (54.6)	1965 (55.4)	318 (56.9)	102 (67.1)
Male	5603 (45.8)	2499 (47.4)	1234 (45.4)	1579 (44.6)	241 (43.1)	50 (32.9)
Race and ethnicity						
Black	457 (3.7)	210 (4.0)	101 (3.7)	120 (3.4)	<11^a^	<11^a^
Hispanic	475 (3.9)	197 (3.7)	106 (3.9)	142 (4.0)	<11^a^	<11^a^
White	8857 (72.3)	3729 (70.7)	2013 (74.1)	2605 (73.5)	391 (69.9)	119 (78.3)
Other^b^	2456 (20.1)	1138 (21.6)	496 (18.3)	677 (19.1)	120 (21.5)	25 (16.4)
Follow-up duration, mean (SD), mo	26.9 (2.4)	28.0 (8.6)	27.4 (9.4)	26.2 (9.5)	12.4 (3.9)	27.3 (8.7)
Prior health care utilization^c^						
Procedures	8543 (69.8)	3863 (73.2)	1863 (68.6)	2365 (66.7)	366 (65.5)	86 (56.6)
Imaging	7894 (64.5)	3570 (67.7)	1655 (60.9)	2246 (63.4)	358 (64.0)	65 (42.8)
ED visits	5786 (47.3)	2581 (48.9)	1253 (46.1)	1629 (46.0)	258 (46.2)	65 (42.8)
Hospitalization	5085 (41.5)	2091 (39.6)	1160 (42.7)	1519 (42.9)	254 (45.4)	61 (40.1)
Surgery	>11^a^	260 (4.9)	138 (5.1)	263 (7.4)	33 (5.9)	<11^a^
Serious infections	2381 (19.4)	1266 (24.0)	606 (22.3)	359 (10.1)	119 (21.3)	31 (20.4)
Charlson Comorbidity Index score						
Mean (SD)	1.0 (1.6)	0.9 (1.5)	1.2 (1.8)	1.0 (1.6)	1.1 (1.6)	1.7 (1.8)
1-2	4219 (34.4)	1819 (34.5)	910 (33.5)	1229 (34.5)	187 (33.4)	74 (48.7)
3-4	1029 (8.4)	375 (7.1)	298 (11.0)	264 (7.4)	66 (11.8)	26 (17.1)
≥5	563 (4.6)	185 (3.5)	179 (6.6)	163 (4.6)	25 (4.5)	11 (7.2)
Comorbidity						
Diabetes	1290 (10.5)	509 (9.7)	353 (13.0)	329 (9.3)	71 (12.7)	28 (18.4)
Hypertension	3853 (31.5)	1503 (28.5)	1001 (36.9)	1087 (30.7)	206 (36.9)	56 (36.8)
Obesity	1782 (14.6)	706 (13.4)	414 (15.2)	518 (14.6)	112 (20.0)	32 (21.1)
CKD	752 (6.1)	248 (4.7)	231 (8.5)	217 (6.1)	45 (8.1)	11 (7.2)
Smoking	>11^a^	231 (4.4)	107 (3.9)	395 (11.1)	15 (2.7)	<11^a^
Prior CAD	99 (0.8)	21 (0.4)	14 (0.5)	60 (1.7)	<11^a^	<11^a^
Hyperlipidemia	2768 (22.6)	1120 (21.2)	717 (26.4)	734 (20.7)	154 (27.5)	43 (28.3)
IBD-related and other medication use						
Prior steroid use^d^	6122 (50.0)	2578 (48.9)	1391 (51.2)	1742 (49.2)	297 (53.1)	114 (75.0)
Concomitant steroid use^e^	3784 (30.9)	1756 (33.3)	806 (29.7)	1024 (28.9)	134 (24.0)	64 (42.1)
Prior IMM use^d^	2670 (21.8)	1070 (20.3)	627 (23.1)	836 (23.6)	91 (16.3)	46 (30.3)
Concomitant IMM use^e^	1980 (16.2)	1058 (20.1)	375 (13.8)	480 (13.5)	40 (7.2)	27 (17.8)
Concomitant opiate use	2231 (18.2)	924 (17.5)	501 (18.4)	668 (18.8)	90 (16.1)	48 (31.6)
Prior TNF-α antagonist exposure	2071 (16.9)	0	689 (25.4)	1222 (34.5)	112 (20.0)	48 (31.6)
Prior VDZ exposure	>11^a^	15 (0.3)	0	33 (0.9)	<11^a^	0
Concomitant VDZ use	163 (1.3)	<11^a^	147 (5.4)	<11^a^	<11^a^	0
Prior JAK inhibitor use	36 (0.3)	<11^a^	<11^a^	<11^a^	<11^a^	0
Prior IL-12/23 use	533 (4.4)	155 (2.9)	203 (7.5)	0	139 (24.9)	36 (23.7)
Prior anticoagulant use	>11^a^	233 (4.4)	160 (5.9)	210 (5.9)	41 (7.3)	<11^a^
Prior hormonal contraceptive use	1152 (9.4)	548 (10.4)	207 (7.6)	340 (9.6)	42 (7.5)	15 (9.9)

Abbreviations: CAD, coronary artery disease; CKD, chronic kidney disease; ED, emergency department; IBD, inflammatory bowel disease; IL, interleukin; IMM, immunomodulator; JAK, Janus kinase inhibitors; TNF, tumor necrosis factor; VDZ, vedolizumab.

^a^ Cell suppression based on Optum Labs’ cell-size suppression rules; numbers less than 11 were masked to protect patient confidentiality.

^b^ Categories include American Indian or Alaska Native, Asian, Pacific Islander, and individuals not captured in predefined categories.

^c^ 12 Months before starting the biologic therapy.

^d^ Indicates usage from 1 year prior to the index to 30 days before the index date (−365 to −30 days).

^e^ Indicates usage within 30 days before (−30 days) or after (+30 days) the index date.

### Serious Infections

**Figure 1.**
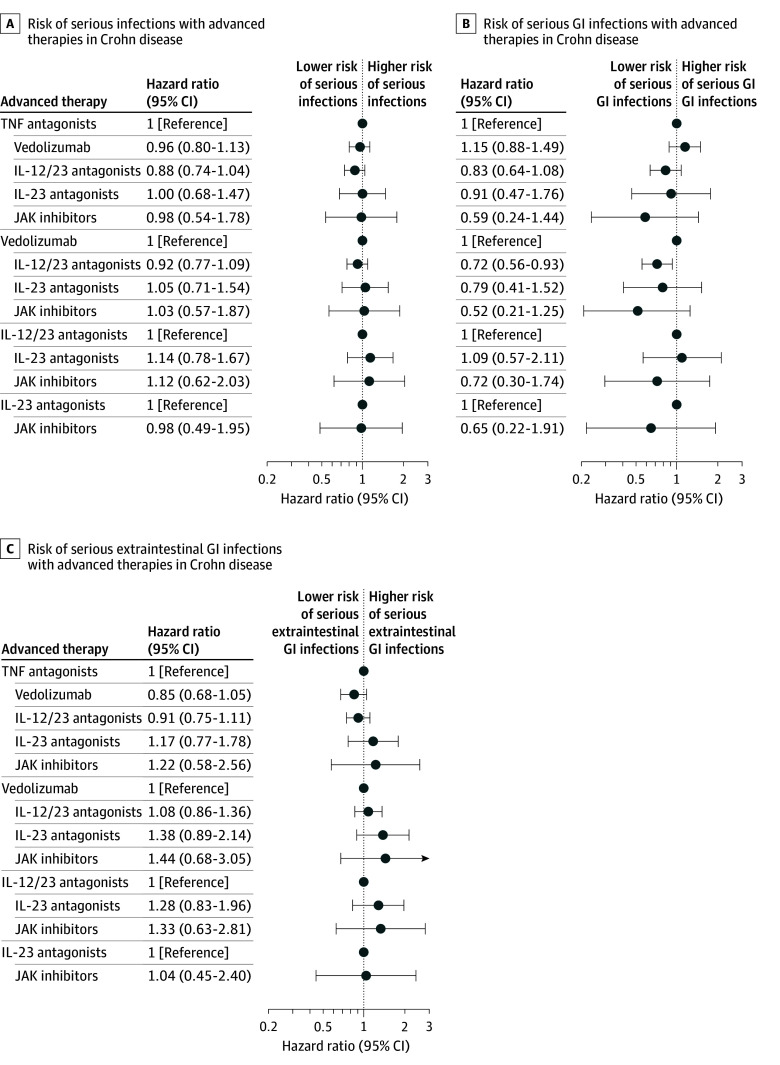
Forest Plot of Comparative Risk of Serious Infections, Serious Gastrointestinal (GI) Infections, and Extraintestinal GI Infections With Different Advanced Therapies in Patients With Crohn Disease IL-12/23 indicates interleukin-12/23p40; IL-23, interleukin-23p19; JAK, Janus kinase inhibitors; TNF, tumor necrosis factor-α.

After starting therapy, the incidence rate of serious infections per 100 person-years by advanced therapy was 5.52 (95% CI, 5.02-6.01) for TNF antagonists, 6.65 (95% CI, 5.88-7.44) for anti-integrins, 5.46 (95% CI, 4.86-6.07) for IL-12/23p40 antagonists, 9.02 (95% CI, 6.38-11.89) for IL-23p19 antagonists, and 8.81 (95% CI, 4.95-13.22) for JAK inhibitors (Table 2). After controlling for confounding variables using IPW analysis (eFigure 1A in Supplement 1), additionally adjusting for age, which was unbalanced after IPW and competing risk of mortality, as compared with TNF antagonists, no significant differences were observed in the risk of serious infections with anti-integrins (HR, 0.96 [95% CI, 0.80-1.13]), IL-12/23p40 antagonists (HR, 0.88 [95% CI, 0.74-1.04]), IL-23p19 antagonists (HR, 1.00 [95% CI, 0.68-1.47]), and JAK inhibitors (HR, 0.98 [95% CI, 0.54-1.78]). Similarly, no significant differences were observed in the overall risk of serious infections between different advanced therapies in patients with CD (risankizumab vs ustekinumab: HR, 1.14 [95% CI, 0.78-1.67]). Cumulative incidence curves and HRs comparing risk of serious infections with each advanced therapy vs others are shown in Figure 1A and eFigure 2A in Supplement 1. Similar results were observed in the sensitivity analysis that excluded patients with rheumatoid arthritis, psoriatic arthritis, or psoriasis at baseline (eTable 1 in Supplement 1).

**Table 2.** Risk of Serious Infections, Venous Thromboembolism, and Major Adverse Cardiovascular Events Comparing Different Advanced Therapies in Patients With Crohn Disease

Advanced therapy	Incidence rate per 100 person-years (95% CI)				
	Serious infection			Venous thromboembolism	Major adverse cardiovascular events
	Overall	Gastrointestinal	Extraintestinal		
TNF-α antagonists	5.52 (5.02-6.01)	2.42 (2.10-2.75)	3.09 (2.72-3.46)	0.90 (0.71-1.10)	0.68 (0.51-0.85)
Anti-integrin	6.65 (5.88-7.44)	3.39 (2.85-3.95)	3.25 (2.71-3.81)	1.19 (0.88-1.52)	1.08 (0.79-1.39)
IL-12/23p40 antagonists	5.46 (4.86-6.07)	2.37 (1.98-2.77)	3.09 (2.63-3.55)	1.14 (0.87-1.42)	0.74 (0.52-0.95)
IL-23p19 antagonists	9.02 (6.38-11.89)	2.86 (1.32-4.62)	6.16 (3.96-8.58)	2.33 (1.06-3.82)	1.49 (0.43-2.76)
JAK inhibitors	8.81 (4.95-13.22)	3.85 (1.10-7.16)	4.96 (2.20-8.26)	1.04 (0-2.60)	0

Abbreviations: IL, interleukin; JAK, Janus kinase; TNF, tumor necrosis factor.

The most common gastrointestinal serious infections were perianal abscesses and *Clostridioides difficile* infection, and the most common extraintestinal serious infections were sepsis, pneumonia, and urinary tract infection. On specifically examining the risk of gastrointestinal serious infections, we observed that IL-12/23p40 antagonists were associated with lower risk of gastrointestinal serious infections compared with anti-integrins (HR, 0.72 [95% CI, 0.56-0.93]), while no significant differences were observed among the other agents (Figure 1B). There were no significant differences in risk of extraintestinal serious infections between different advanced therapies in patients with CD (Figure 1C).

### VTE

**Figure 2.**
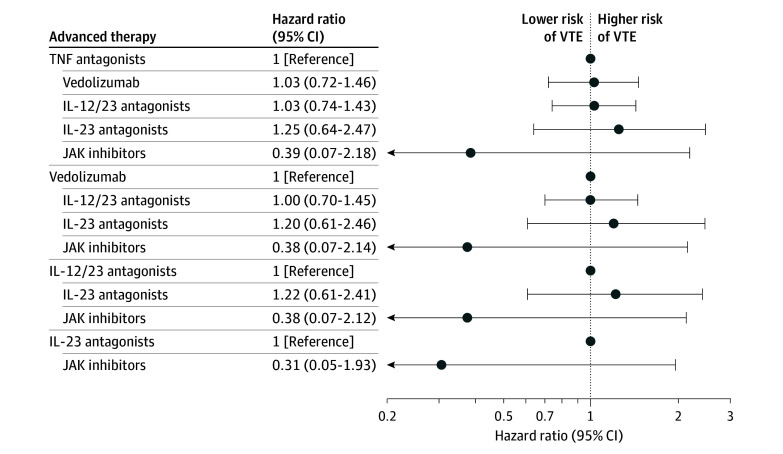
Forest Plot of Comparative Risk of Venous Thromboembolism (VTE) With Different Advanced Therapies in Patients With Crohn Disease IL-12/23 indicates interleukin-12/23p40; IL-23, interleukin-23p19; JAK, Janus kinase inhibitors; TNF, tumor necrosis factor-α.

After starting therapy, the incidence rate of VTE per 100 person-years by advanced therapy was 0.90 (95% CI, 0.71-1.10) for TNF antagonists, 1.19 (95% CI, 0.88-1.52) for anti-integrins, 1.14 (95% CI, 0.87-1.42) for IL-12/23p40 antagonists, 2.33 (95% CI, 1.06-3.82) for IL-23p19 antagonists, and 1.04 (95% CI, 0-2.60) for JAK inhibitors (Table 2). After controlling for confounding variables using IPW analysis (eFigure 1B in Supplement 1), additionally adjusting for age, which was unbalanced after IPW and competing risk of mortality, as compared with TNF antagonists, no significant differences were observed in the risk of VTE with anti-integrins (HR, 1.03 [95% CI, 0.72-1.45]), IL-12/23p40 antagonists (HR, 1.03 [95% CI, 0.75-1.41]), IL-23p19 antagonists (HR, 1.25 [95% CI, 0.65-2.43]), and JAK inhibitors (HR, 0.39 [95% CI, 0.07-2.18]). No significant differences were observed in the risk of VTE between different advanced therapies in patients with CD. Cumulative incidence curves and HRs comparing risk of VTE with each advanced therapy vs others are shown in Figure 2 and eFigure 2B in Supplement 1. Similar results were observed in the sensitivity analysis that excluded patients with rheumatoid arthritis, psoriatic arthritis, or psoriasis at baseline (eTable 1 in Supplement 1). On sensitivity analysis, limiting VTE events during hospitalization or emergency department visit, JAK inhibitors were associated with lower risk of VTE compared with TNF antagonists, anti-integrins, IL-12/23p40 antagonists, and IL-23p19 antagonists (eTable 2 in Supplement 1).

### MACE

**Figure 3.**
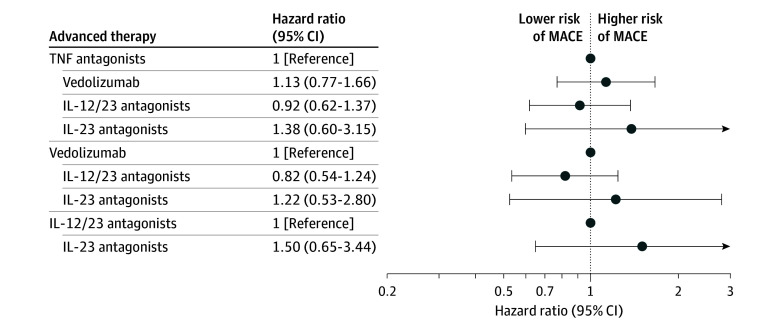
Forest Plot of Comparative Risk of Major Adverse Cardiovascular Events (MACE) With Different Advanced Therapies in Patients With Crohn Disease IL-12/23 indicates interleukin-12/23p40; IL-23, interleukin-23p19; JAK, Janus kinase inhibitors; TNF, tumor necrosis factor-α.

After starting therapy, the incidence rate of MACE per 100 person-years by advanced therapy was 0.68 (95% CI, 0.51-0.85) for TNF antagonists, 1.08 (95% CI, 0.79-1.39) for anti-integrins, 0.74 (95% CI, 0.52-0.95) for IL-12/23p40 antagonists, and 1.49 (95% CI, 0.43-2.76) for IL-23p19 antagonists; no MACE events were seen in patients treated with JAK inhibitors (Table 2). After controlling for confounding variables using IPW analysis (eFigure 1C in Supplement 1) and competing risk of mortality, as compared with TNF antagonists, no significant differences were observed in the risk of MACE with anti-integrins (HR, 1.13 [95% CI, 0.77-1.65]), IL-12/23p40 antagonists (HR, 0.92 [95% CI, 0.62-1.36]), and IL-23p19 antagonists (HR, 1.38 [95% CI, 0.60-3.15]). No significant differences were observed in the risk of MACE between different advanced therapies in patients with CD. Cumulative incidence curves and HRs comparing risk of MACE with each advanced therapy vs others are shown in Figure 3 and eFigure 2C in Supplement 1. Similar results were observed in the sensitivity analysis that excluded patients with rheumatoid arthritis, psoriatic arthritis, or psoriasis at baseline (eTable 1 in Supplement 1).

## Discussion

In this comparative effectiveness research study of 12 245 patients with CD who were treated with TNF antagonists, anti-integrin agents (vedolizumab), IL-12/23p40 antagonists (ustekinumab), IL-23p19 antagonists (primarily risankizumab), and JAK inhibitors (upadacitinib), we made several key observations on the comparative safety of advanced therapies in patients with CD. First, across all pair-wise comparisons, there were no significant differences in the risk of serious infections with different agents, including no difference between TNF antagonists and IL-12/23p40 antagonists (ustekinumab) or IL-23p19 antagonists, between IL-12/23p40 antagonists and selective IL-23p19 antagonists, and between TNF antagonists and JAK inhibitors. We observed a lower risk of serious infections of gastrointestinal origin (possibly associated with disease-related complication) with IL-12/23p40 antagonists vs vedolizumab. Second, the incidence of VTE in patients with CD was low and comparable across all advanced therapies, including JAK inhibitors, over a mean follow-up of 27 months. Third, the incidence of MACE in patients with CD was also low and similar across all advanced therapies, including anti-ILs and JAK inhibitors. Overall, these findings on the comparative safety of different advanced therapies in patients with CD are reassuring by not identifying a significantly increased hazard of serious infections, VTE, or MACE with any particular class of advanced therapies. These findings support clinical decision-making on choice of advanced therapies for individual patients to be driven primarily by comparative treatment effectiveness rather than driven by concerns of serious adverse events for most patients with CD. Effective treatment may mitigate risks of disease-related complications, decrease corticosteroid use, and improve patients’ functional status in decreasing risk of infections.^23^

Recent head-to-head RCTs have suggested comparable efficacy of ustekinumab and adalimumab and superiority of IL-23p19 antagonists (risankizumab and guselkumab) over ustekinumab, particularly in patients with prior TNF antagonist exposure, without any differences in key safety end points.^11,24,25^ Consequently, there is considerable interest in positioning IL-23p19 antagonists and IL-12/23p40 antagonists as first-line therapies for most patients with CD. However, to date, there has been a paucity of comparative safety data comparing IL-23p19 antagonists vs IL-12/23p40 antagonists vs TNF antagonists. In a multicenter, propensity score–matched electronic health record–based registry, Singh and colleagues^26^ observed that ustekinumab may be associated with 64% lower risk of serious infections compared with TNF antagonists, with a 1-year risk of serious infection being 2.5% vs 6.9%. However, the study was limited in only capturing hospitalizations occurring within the participating hospitals, relied only on medication prescriptions in the electronic health record, and was unable to verify medication dispensing and compliance. In a claims-based analysis comparing risk of serious infections between ustekinumab and TNF antagonists, Cheng and colleagues^27^ did not observe a significant difference with a fairly similar point estimate as our study (HR, 0.84 vs 0.88 in the current study). In a nationwide cohort study in Sweden, Forss and colleagues^28^ similarly observed no significant differences in the risk of serious infections between ustekinumab and TNF antagonists (incidence rate, 4.1 vs 5.0 per 100 person-years; HR, 0.78 [95% CI, 0.59-1.03]). To our knowledge, the present study is the first observational study comparing the risk of serious infections of IL-23p19 antagonists with TNF antagonists or with IL-12/23p40 antagonists. In a recent meta-analysis of 5 RCTs comparing IL-23p19 antagonists with IL-12/23p40 antagonists, Dziegielewski and colleagues^25^ observed no significant difference in the risk of serious infections (1.8% vs 3.5%; relative risk, 0.56 [95% CI, 0.25-1.24]). In our analysis, similarly, there was no significant difference in the risk of serious infections between IL-23p19 antagonists and IL-12/23p40 antagonists, including gastrointestinal and extraintestinal infections. Thus, while there may be theoretical safety benefits with selective blockade of IL-23, current evidence does not suggest any significant differences in risk of serious infections compared with IL-12/23p40 antagonists or TNF antagonists.

The ORAL Surveillance (Safety Study of Tofacitinib Versus Tumor Necrosis Factor [TNF] Inhibitor in Subjects With Rheumatoid Arthritis) trial, demonstrating a higher risk of VTE and MACE with tofacitinib vs TNF antagonists in older adults with rheumatoid arthritis, and subsequent regulatory decisions to restrict the use of all JAK inhibitors across all indications have brought concerns about VTE and MACE to the fore in patients with IBD.^13^ In a network meta-analysis of 36 RCTs of all licensed biologics and small molecules for patients with IMIDs, Mattay et al^29^ observed increased risk of MACE with TNF antagonists, IL-12/23p40 antagonists, and JAK inhibitors compared with placebo but no difference in the risk between difference advanced therapies. Multiple observational studies have not replicated findings from the ORAL Surveillance trial, with most suggesting no significant difference in VTE and MACE between JAK inhibitors and TNF antagonists across IMIDs.^30,31^ In our analysis, the number of patients treated with JAK inhibitors was small (n = 152), the incidence of VTE was low (1.0 per 100 person-years), and none of the patients experienced a MACE event. We observed a universally low incidence of VTE and MACE among patients with CD who were treated with advanced therapies. Similar to the network meta-analysis of RCTs,^29^ the risk was comparable across all agents.

### Strengths and Limitations

Our study has important strengths. These include a large cohort of more than 12 000 patients with CD who were treated with advanced therapies, simultaneous evaluation of the risk of key safety events with all approved advanced therapies in patients with CD, use of robust IPW to balance treatment selection with multinomial propensity scores, and accounting for competing risk of mortality.

We acknowledge several important limitations to our study. First, as an administrative claims database study, we did not have access to subjective or objective measures of disease activity or endoscopy reports and did not have accurate details of disease location and behavior. Second, as with any observational study, we cannot rule out residual confounding, especially those due to treatment selection. For example, some risk factors for thrombotic and atherosclerotic diseases like dyslipidemia, smoking, and obesity may be inadequately captured in administrative databases based on claims codes. However, our analytical approach, with a new-user cohort design, accounting for key confounders including corticosteroid exposure and hospitalization, which may serve as a surrogate of disease activity, provides some protection against bias. Third, the incidence of some events like VTE and MACE was low, which decreased confidence in some effect estimates. Median duration of follow-up in our study’s cohort was 2.3 years compared with 4 years in the ORAL Surveillance trial. Fourth, the number of patients treated with JAK inhibitors was relatively small (n = 152), which limited the precision of comparative risk estimates, particularly for rare outcomes such as VTE and MACE. Similarly, risankizumab received US Food and Drug Administration approval in 2022, resulting in a smaller sample size and shorter follow-up duration.

## Conclusions

In this comparative effectiveness research study of more than 12 000 patients with CD who were treated with all approved advanced therapies between 2016 to 2022 using a large administrative claims database, the findings suggest that the safety of all agents for key events such as serious infections, VTE, and MACE was similar. The putative safety of IL-23p19 antagonists compared with TNF antagonists and IL-12/23p40 antagonists should be evaluated more comprehensively to allow informed clinical decision-making. The study’s findings of a broadly comparable safety profile among current treatment options support individualized therapy selection based on clinical and patient-centered factors, with a greater emphasis on effectiveness.

## Notes

Supplement 1. **eTable 1. **Incidence Rates and Hazard Ratios for Serious Infections, Venous Thromboembolism, and Major Adverse Cardiovascular Events Comparing Advanced Therapies in Patients With Crohn Disease: Sensitivity Analysis Excluding Patients With Rheumatoid Arthritis, Psoriatic Arthritis, or Psoriasis at Baseline **eTable 2. **Sensitivity Analysis—Comparative Risk of Venous Thromboembolism With Different Advanced Therapies in Patients With Crohn Disease, When VTE Was Defined Based on Events During Inpatient or Emergency Room Visit **eFigure 1. **Covariate Balance Plot for (A) Serious Infections, (B) Venous Thromboembolism, and (C) Major Adverse Cardiovascular Events **eFigure 2.** Cumulative Incidence Curves Comparing the Risk of (A) Serious Infections, (B) Venous Thromboembolism, and (C) Major Adverse Cardiovascular Events in Patients With CD

Supplement 2.
Data Sharing Statement
