# Supplementary material for: Comparative Safety of Advanced Therapies for Crohn Disease
Source: JAMA Netw Open. 2026 Feb 6;9(2):e2557922. doi: 10.1001/jamanetworkopen.2025.57922 (PMC12881986; doi:10.1001/jamanetworkopen.2025.57922)
Supplement: Supplement 1. — eTable 1. Incidence Rates and Hazard Ratios for Serious Infections, Venous Thromboembolism, and Major Adverse Cardiovascular Events Comparing Advanced Therapies in Patients With Crohn Disease: Sensitivity Analysis Excluding Patients With Rheumatoid Arthritis, Psoriatic Arthritis, or Psoriasis at Baseline eTable 2. Sensitivity Analysis—Comparative Risk of Venous Thromboembolism With Different Advanced Therapies in Patients With Crohn Disease, When VTE Was Defined Based on Events During Inpatient or Emergency Room Visit eFigure 1. Covariate Balance Plot for (A) Serious Infections, (B) Venous Thromboembolism, and (C) Major Adverse Cardiovascular Events eFigure 2. Cumulative Incidence Curves Comparing the Risk of (A) Serious Infections, (B) Venous Thromboembolism, and (C) Major Adverse Cardiovascular Events in Patients With CD [file jamanetwopen-e2557922-s001.pdf]

Supplemental Online Content

Park S-k, Ahuja D, Yeh, K-H, et al. Comparative safety of advanced therapies in patients with Crohn disease. *JAMA Netw Open*. 2026;9(2):e2557922. doi:10.1001/jamanetworkopen.2025.57922

eTable 1. Incidence Rates and Hazard Ratios for Serious Infections, Venous Thromboembolism, and Major Adverse Cardiovascular Events Comparing Advanced Therapies in Patients With Crohn Disease: Sensitivity Analysis Excluding Patients With Rheumatoid Arthritis, Psoriatic Arthritis, or Psoriasis at Baseline
eTable 2. Sensitivity Analysis—Comparative Risk of Venous Thromboembolism With Different Advanced Therapies in Patients With Crohn Disease, When VTE Was Defined Based on Events During Inpatient or Emergency Room Visit
eFigure 1. Covariate Balance Plot for (A) Serious Infections, (B) Venous Thromboembolism, and (C) Major Adverse Cardiovascular Events
eFigure 2. Cumulative Incidence Curves Comparing the Risk of (A) Serious Infections, (B) Venous Thromboembolism, and (C) Major Adverse Cardiovascular Events in Patients With CD

This supplemental material has been provided by the authors to give readers additional information about their work.

© 2026 Park S-k et al. *JAMA Network Open*.

eTable 1. Incidence Rates and Hazard Ratios for Serious Infections, Venous Thromboembolism, and Major Adverse Cardiovascular Events Comparing Advanced Therapies in Patients With Crohn Disease: Sensitivity Analysis Excluding Patients With Rheumatoid Arthritis, Psoriatic Arthritis, or Psoriasis at Baseline

	Serious infection			VTE	MACE
	**Overall**	**Gastrointestinal**	**Extra-intestinal**		
**Incidence rate [95% CI], per 100 person-years**					
**TNF antagonists**	5.24 [4.75 - 5.74]	2.43 [2.10 - 2.77]	2.81 [2.45 - 3.18]	0.87 [0.68 - 1.07]	0.59 [0.43 - 0.75]
**Anti-integrin**	6.51 [5.75 - 7.30]	3.35 [2.80 - 3.93]	3.16 [2.63 - 3.71]	1.19 [0.87 - 1.53]	1.07 [0.77 - 1.39]
**IL-12/23p40 antagonists**	5.47 [4.86 - 6.10]	2.41 [2.00 - 2.84]	3.06 [2.60 - 3.54]	1.12 [0.87 - 1.40]	0.71 [0.49 - 0.93]
**IL-23p19 antagonists**	8.68 [6.10 - 11.50]	3.05 [1.41 - 4.93]	5.63 [3.52 - 7.98]	2.27 [0.91 - 3.85]	1.36 [0.45 - 2.50]
**JAK inhibitors**	8.87 [3.33 – 15.53]	4.44 [1.11 - 8.87]	4.44 [1.11 - 8.87]	0.00	0.00
**Hazard ratio [95% CI]**					
**TNF antagonists (ref)**	1	1	1	1	**1**
Anti-integrin	1.02 [0.86 - 1.22]	1.13 [0.86 - 1.47]	0.91 [0.73 - 1.13]	1.08 [0.76 - 1.55]	1.25 [0.81 - 1.94]
IL-12/23p40 antagonists	0.95 [0.80 - 1.14]	0.83 [0.63 - 1.10]	0.99 [0.81 - 1.22]	1.06 [0.76 - 1.48]	0.93 [0.58 - 1.47]
IL-23p19 antagonists	0.98 [0.65 - 1.47]	0.83 [0.43 - 1.62]	1.24 [0.79 - 1.96]	1.31 [0.65 - 2.63]	1.89 [0.74 - 4.86]
JAK inhibitors	0.99 [0.42 – 2.34]	0.65 [0.21 -2.08]	1.04 [0.35 - 3.09]	NA	NA
**Anti-integrin (ref)**	1	1	1	1	1
IL-12/23p40 antagonists	0.93 [0.78 - 1.11]	**0.74 [0.57 - 0.95]**	1.09 [0.87 - 1.39]	0.98 [0.68 - 1.41]	0.74 [0.48 - 1.16]
IL-23p19 antagonists	0.95 [0.63 - 1.43]	0.74 [0.38 - 1.43]	1.37 [0.86 - 2.18]	1.21 [0.59 - 2.50]	1.51 [0.60 - 3.80]
JAK inhibitors	0.96 [0.41 - 2.28]	0.58 [0.18 - 1.83]	1.14 [0.38 - 3.41]	NA	NA

© 2026 Park S-k et al. *JAMA Network Open*.

**IL-12/23p40 antagonists (ref)**	1	1	1	1	1
IL-23p19 antagonists	1.02 [0.68 - 1.53]	1.00 [0.51 – 1.93]	1.25 [0.79 - 1.98]	1.24 [0.61 - 2.49]	2.04 [0.81 - 5.16]
JAK inhibitors	1.03 [0.44 - 2.45]	0.78 [0.25 - 2.48]	1.04 [0.35 - 3.11]	NA	NA
**IL-23p19 antagonists**	1	1	1	1	1
JAK inhibitors	1.01 [0.40 - 2.56]	0.79 [0.22 - 2.84]	0.83 [0.26 - 2.68]	NA	NA

[Abbreviations: GI = Gastrointestinal VTE=venous thromboembolism, MACE=major adverse cardiovascular events CI=confidence interval, JAK=Janus kinase; py=person-years, TNF=tumor necrosis factor, IL=interleukin, NA= not available]

© 2026 Park S-k et al. *JAMA Network Open*.

eTable 2. Sensitivity Analysis—Comparative Risk of Venous Thromboembolism With Different Advanced Therapies in Patients With Crohn Disease, When VTE Was Defined Based on Events During Inpatient or Emergency Room Visit

Hazard Ratio (95% CI)	
**TNF antagonists (reference)**	**1.0**
Anti-integrin	1.15 [0.72 - 1.82]
IL-12/23p40 antagonists	0.94 [0.60 - 1.47]
IL-23p19 antagonists	1.26 [0.48 – 3.34]
JAK inhibitors	**0.10 [0.01 – 0.74]**
**Anti-integrin (ref)**	**1.0**
IL-12/23p40 antagonists	0.82 [0.49 - 1.37]
IL-23p19 antagonists	1.10 [0.41 - 2.99]
JAK inhibitors	**0.09 [0.01 - 0.66]**
**IL-12/23p40 antagonists (ref)**	**1.0**
IL-23p19 antagonists	1.34 [0.50 - 3.62]
JAK inhibitors	**0.11 [0.01 - 0.80]**
**IL-23p19 antagonists**	**1.0**
JAK inhibitors	**0.08 [0.01 - 0.77]**

© 2026 Park S-k et al. *JAMA Network Open*.

eFigure 1. Covariate Balance Plot for (A) Serious Infections, (B) Venous Thromboembolism, and (C) Major Adverse Cardiovascular Events

(A) Serious infections

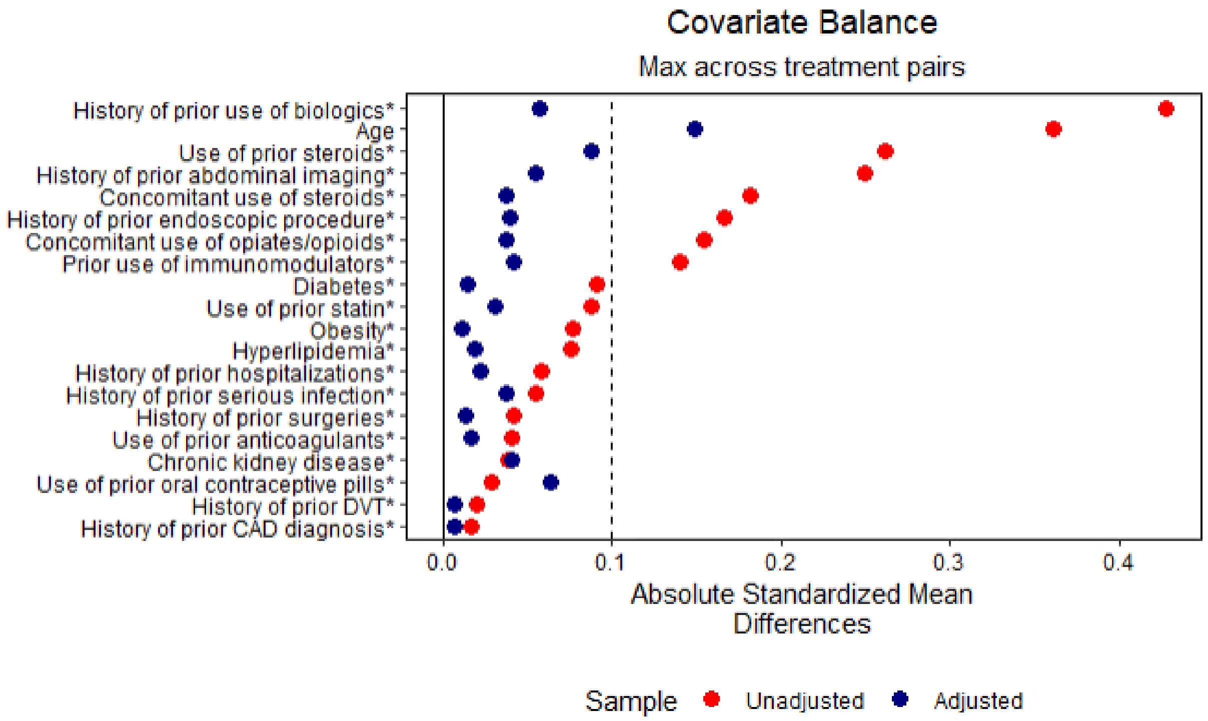

© 2026 Park S-k et al. *JAMA Network Open*.

(B) Venous thromboembolism

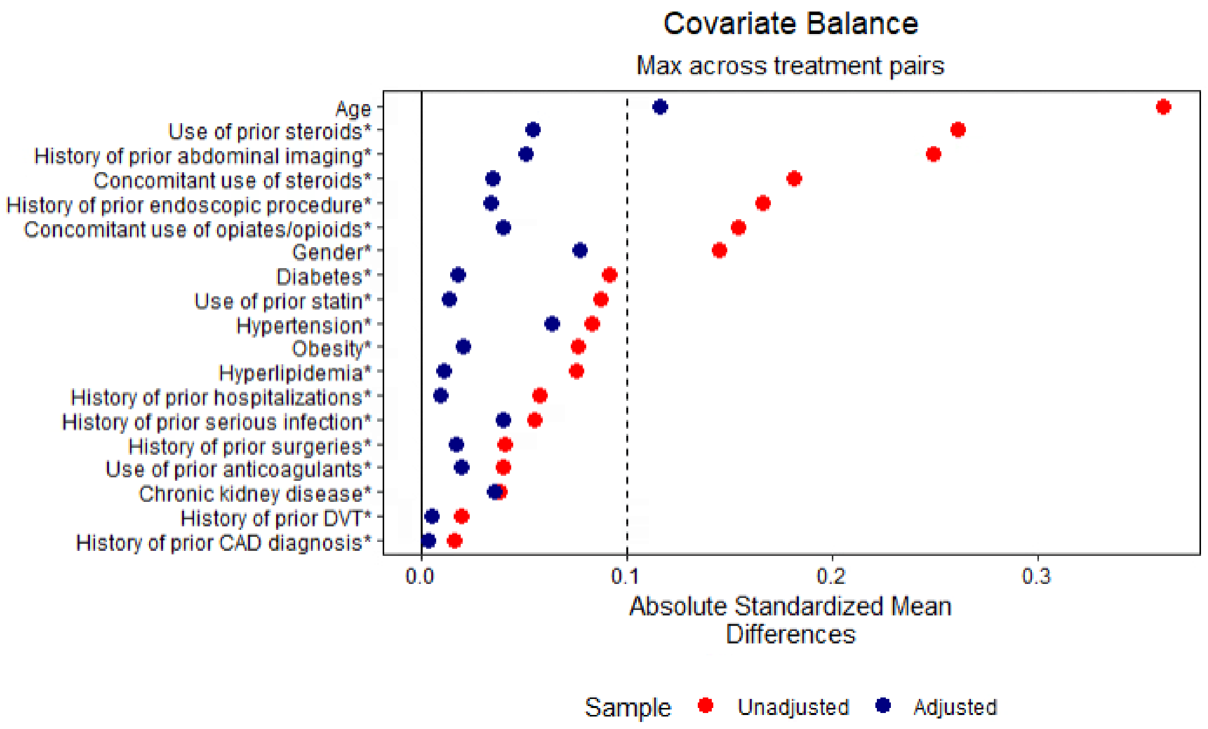

© 2026 Park S-k et al. *JAMA Network Open*.

(C) Major adverse cardiovascular events

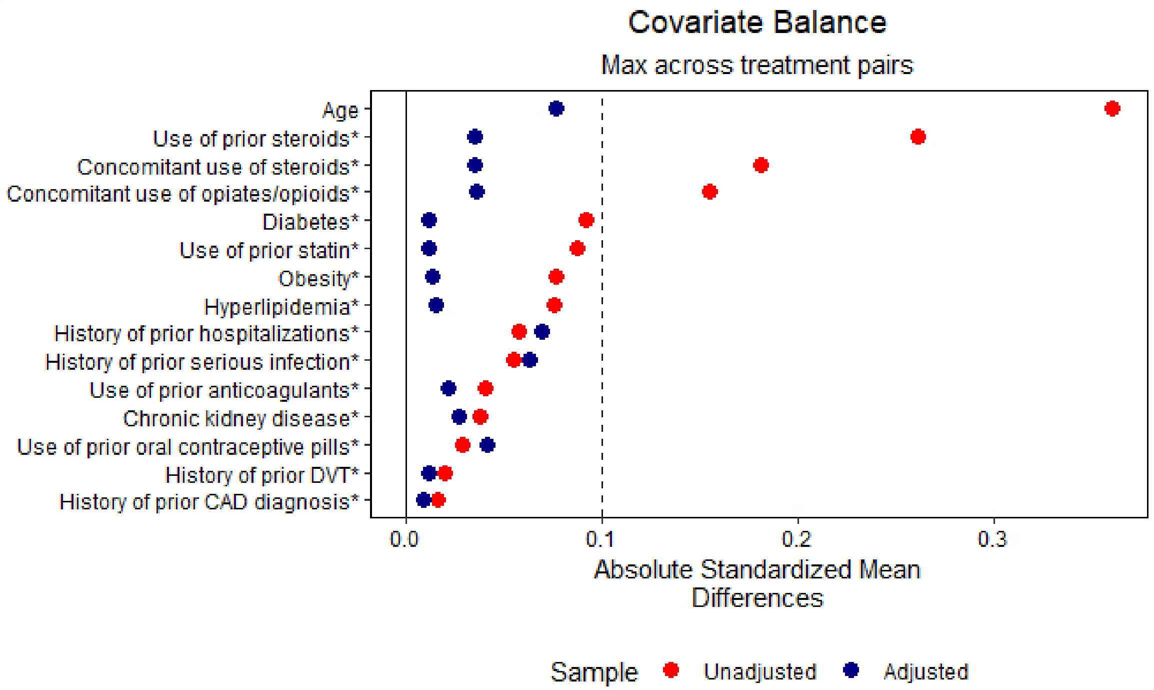

© 2026 Park S-k et al. *JAMA Network Open*.

eFigure 2. Cumulative Incidence Curves Comparing the Risk of (A) Serious Infections, (B) Venous Thromboembolism, and (C) Major Adverse Cardiovascular Events in Patients With CD

(A) Serious infections

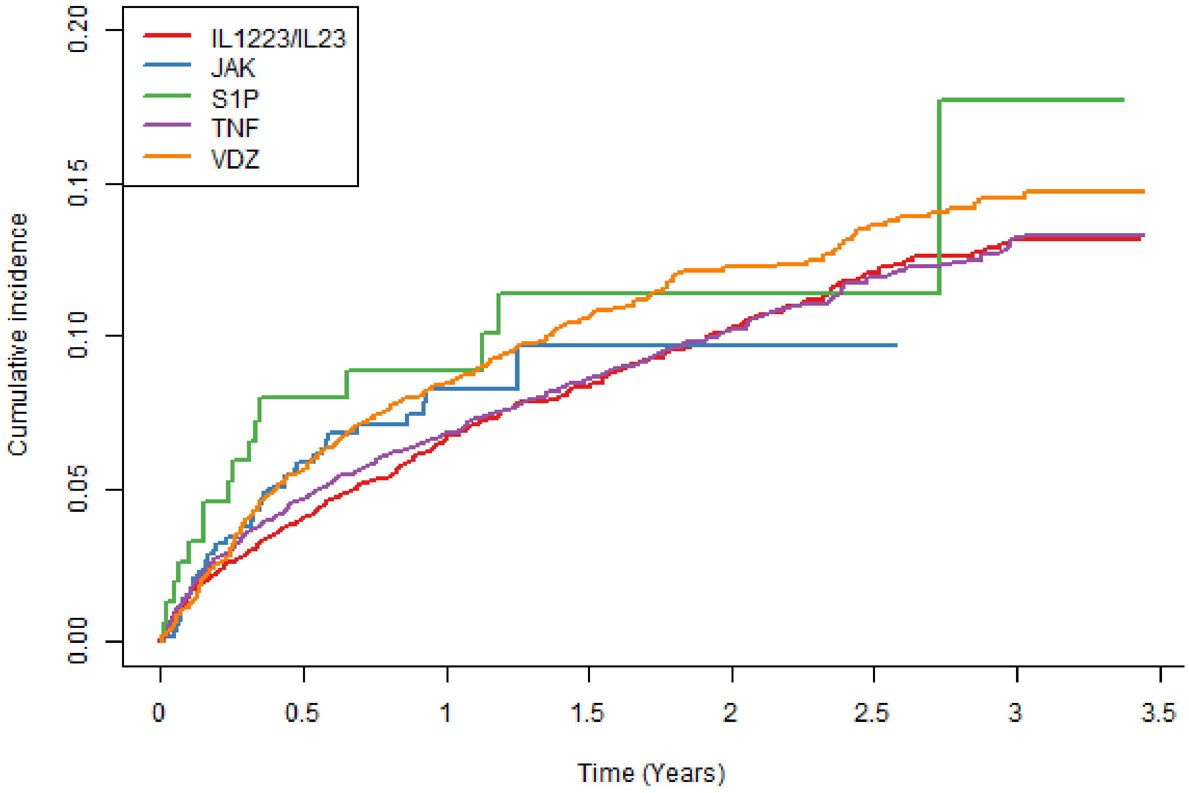

© 2026 Park S-k et al. *JAMA Network Open*.

(B) Venous thromboembolism

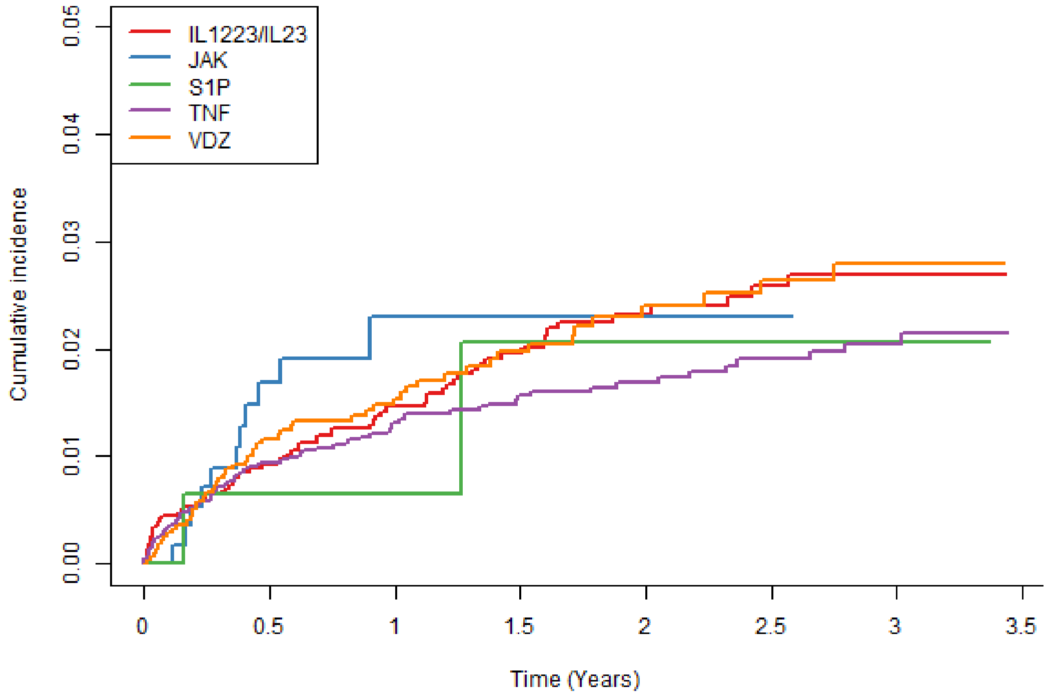

© 2026 Park S-k et al. *JAMA Network Open*.

(C) Major adverse cardiovascular events

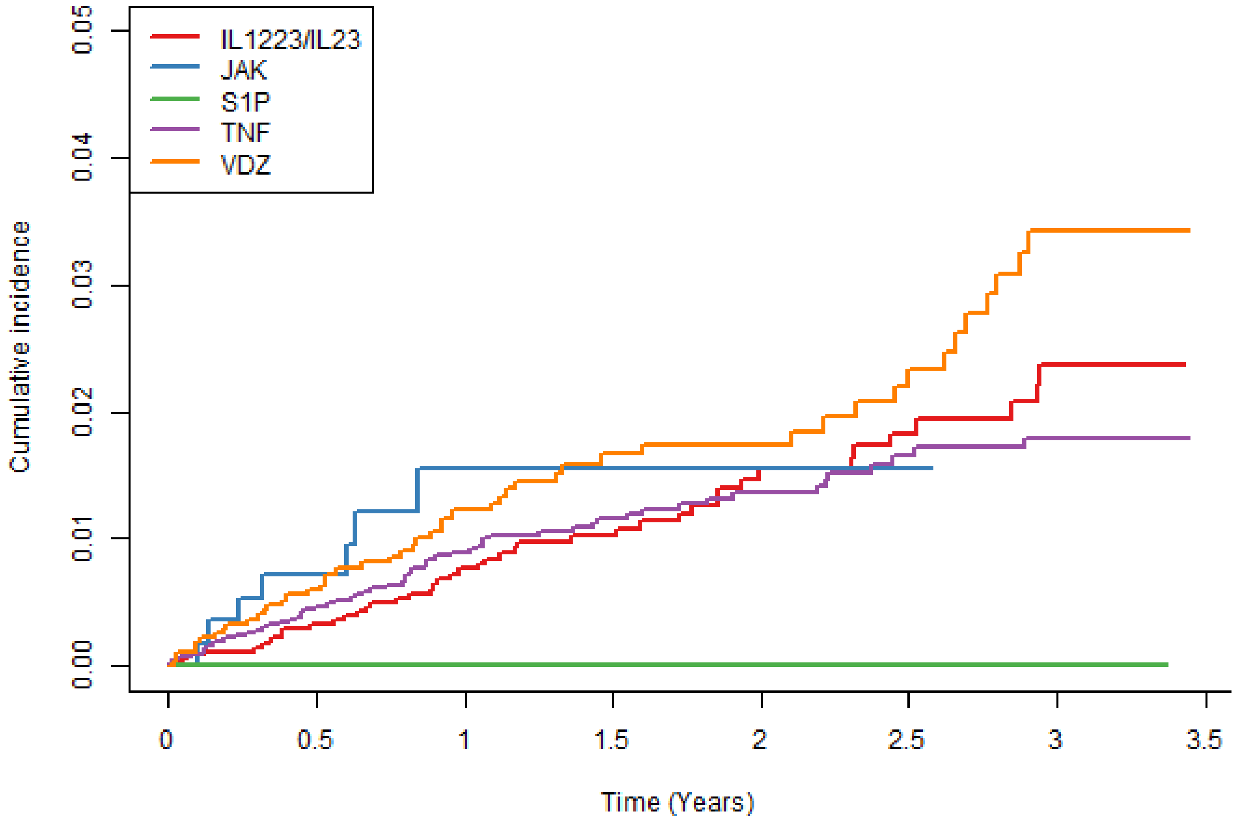

© 2026 Park S-k et al. *JAMA Network Open*.
