# Supplementary material for: Comparative Safety of Advanced Therapies for Crohn Disease
Source: JAMA Netw Open. 2026 Feb 6;9(2):e2557922. doi: 10.1001/jamanetworkopen.2025.57922 (PMC12881986; doi:10.1001/jamanetworkopen.2025.57922)
Supplement: Supplement 2. — Data Sharing Statement [file jamanetwopen-e2557922-s002.pdf]

Data Sharing Statement

Park. Comparative Safety of Advanced Therapies in Patients With Crohn Disease. *JAMA Netw Open*. Published February 06, 2026. doi:10.1001/jamanetworkopen.2025.57922

Data
Data available: No
